# Functional and Structural Analysis of the Central Retina According to the Fundus Autofluorescence Pattern in Patients with Retinitis Pigmentosa

**DOI:** 10.3390/diagnostics16040597

**Published:** 2026-02-17

**Authors:** Marta P. Wiącek, Kinga Skorupińska, Miszela Kałachurska, Anna Machalińska

**Affiliations:** First Department of Ophthalmology, Pomeranian Medical University, 70-204 Szczecin, Poland; marta.wiacek@pum.edu.pl (M.P.W.); kingaskor28@gmail.com (K.S.); miszela.kaachurska@gmail.com (M.K.)

**Keywords:** retinitis pigmentosa, fundus autofluorescence, hyperautofluorescent ring, optical coherence tomography, multifocal electroretinography, ellipsoid zone, macular function, disease progression monitoring

## Abstract

**Background**: This study evaluated morphological and functional differences among eyes with retinitis pigmentosa (RP) classified according to fundus autofluorescence (FAF) patterns. **Methods**: A total of 146 eyes from 73 patients with RP were analysed. Based on FAF imaging, eyes were classified as having regular hyperautofluorescent rings (n = 23), irregular rings (n = 76), or absent rings (n = 47). Participants underwent best-corrected visual acuity (BCVA), contrast sensitivity, 10–2 and 30–2 static perimetry, multifocal electroretinography (mfERG), and optical coherence tomography (OCT). FAF morphometrics included ring diameters and area. **Results**: Eyes with a regular FAF ring demonstrated significantly better visual function than those with irregular or absent rings, including higher BCVA (*p* < 0.001 and *p* = 0.001) and greater contrast sensitivity (both *p* < 0.001). The mfERG amplitude density in the first ring was higher in regular than irregular FAF patterns (*p* = 0.034). Eyes with irregular FAF showed more advanced visual field loss, with lower mean deviation on 10–2 (*p* = 0.042) and 30–2 perimetry (*p* = 0.027). In the regular-ring group, the ellipsoid zone was predominantly intact (*p* = 0.012). The hyperautofluorescent ring area correlated positively with mfERG amplitude density in the first and second rings (Rs = +0.573, *p* = 0.016; Rs = +0.736, *p* = 0.001) and with macular volume (Rs = +0.667, *p* = 0.003). **Conclusions**: FAF patterns reflect central retinal functional and structural impairment in RP. Therefore, incorporating FAF imaging into the diagnostic algorithm is valuable for monitoring disease progression.

## 1. Introduction

Retinitis pigmentosa (RP) represents the most common form of hereditary retinal degeneration. It affects approximately 1 in 5000 individuals worldwide, accounting for more than 1.5 million people. To date, more than 3100 mutations across over 200 genes have been identified as causative of the clinical manifestations of RP. The disease typically begins with impaired night vision and dyschromatopsia, followed by progressive constriction of the peripheral visual field that may eventually lead to tunnel vision. As degeneration extends to the macular region, patients experience a gradual decline in visual acuity, which can ultimately progress to complete blindness. Owing to its genetic heterogeneity and complex pathogenesis, no universally effective therapy or standardized diagnostic algorithm for the early detection of RP progression has yet been established [1,2,3].

Under short-wavelength light, lipofuscin—a byproduct of photoreceptor degradation—exhibits intrinsic fluorescence. This property forms the basis of fundus autofluorescence (FAF) imaging, which enables noninvasive visualization of lipofuscin accumulation within the retinal pigment epithelium (RPE). In eyes affected by RP, a distinctive hyperautofluorescent ring often delineates the boundary between degenerated retinal areas and zones containing functionally preserved photoreceptors. Although the FAF signal is weaker than that observed in fluorescein angiography, FAF imaging is safer, faster, and highly suitable for repeated use in longitudinal follow-up, making it a promising tool for RP diagnosis [4,5]. Moreover, the integration of FAF with other modern imaging modalities might offer a comprehensive approach for evaluating disease progression and retinal integrity in RP patients [1,6].

Despite its growing availability, the precise clinical value of FAF in RP and the specific parameters crucial for interpreting its patterns remain insufficiently defined. Therefore, this study aimed to evaluate morphological and functional differences in eyes with RP classified according to distinct FAF patterns to clarify the diagnostic and monitoring potential of this imaging modality.

## 2. Materials and Methods

### 2.1. Subjects

This cross-sectional observational study included eyes diagnosed with RP on the basis of characteristic clinical presentation and ancillary testing. In selected cases, genetic confirmation of mutations associated with RP was possible. The diagnosis was established from a comprehensive clinical evaluation that included both patient history and ophthalmic examination. Full-field electroretinography (ERG) confirmed the diagnosis in all participants by revealing markedly reduced a- and b-wave amplitudes, prolonged implicit times, or non-recordable responses (2). In addition, disease-causing genetic mutations were identified in selected cases.

All participants provided written informed consent before study enrolment. Individuals with other ocular disorders, including glaucoma, diabetic or vascular retinopathy, a history of ocular trauma or intraocular surgery other than cataract extraction, or significant media opacities were excluded from the study. The research protocol was approved by the Institutional Review Board and conducted in accordance with the Declaration of Helsinki (protocol code KB-006/42/2022).

Each patient underwent a standardized ophthalmic examination. Best-corrected visual acuity (BCVA) was assessed using ETDRS chart, intraocular pressure (IOP) was measured with a Haag-Streit tonometer (Koeniz, Switzerland), and contrast sensitivity was evaluated with the Pelli–Robson chart according to standard procedures [7]. A slit-lamp examination was performed to exclude anterior segment abnormalities. To ensure optimal reproducibility, all visual acuity tests were conducted under standardized dim illumination. The functional assessment included 10-2 and 30-2 white-on-white (W–W) static perimetry using a Humphrey Field Analyser (Zeiss, Dublin, CA, USA). The acquisition procedures for each examination are described in detail in the following subsections.

### 2.2. Fundus Autofluorescence

**Figure 1:**
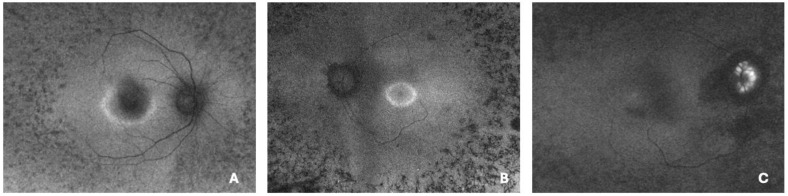
Fundus autofluorescence imaging of eyes with retinitis pigmentosa showing (**A**) an irregular ring, (**B**) a regular hyperautofluorescent ring, and (**C**) an absent ring pattern.

Colour fundus photographs and short-wavelength FAF images were obtained after pharmacological pupil dilation with 1% tropicamide and 10% phenylephrine hydrochloride. Ultrawidefield FAF imaging was performed using the Optos Silverstone system (Marlborough, MA, USA), providing a 200° view of the retina in each eye affected by RP. On the basis of the FAF pattern, all eyes were categorized into three groups: regular hyperautofluorescent rings, irregular rings, and absent rings (Figure 1). In accordance with the classification proposed by Antropoli et al. [8], a regular ring was defined as a well-delineated, elliptic or round area with clearly visible inner and outer borders. By contrast, an irregular ring was characterized by an incomplete or poorly defined border, often deviating from an elliptic shape. Eyes in which no distinct hyperautofluorescent ring was identifiable were classified as absent rings. Importantly, as retinitis pigmentosa may present asymmetrically between fellow eyes, each eye was considered separately for statistical analysis. For quantitative analysis, the largest vertical and horizontal diameters of the ring were measured manually using the system’s built-in software. The internal and external boundaries of the hyperautofluorescent ring were outlined, allowing calculation of the ring area as the difference between the two enclosed regions. All measurements were independently performed by two experienced examiners. In cases where the discrepancy between their results exceeded 10%, a third examiner conducted an additional measurement, and the mean of all three values was used for statistical analysis. Eyes with suboptimal image quality—resulting from poor fixation, insufficient patient cooperation, significant media opacities, or the presence of large floaters—were excluded from the analysis.

### 2.3. Morphometric Measurements of the Macular Region

The macular region was structurally evaluated through spectral-domain optical coherence tomography (SD-OCT; Spectralis, Heidelberg Engineering, Heidelberg, Germany). Prior to image acquisition, the pupils were pharmacologically dilated with 1% tropicamide to ensure optimal image quality. Each OCT examination covered the central 30° of the retina and consisted of up to 100 averaged horizontal and vertical B-scans, each comprising 768 A-scans. All scans were carefully reviewed to confirm the absence of motion artefacts and adequate centration on the fovea. Automated segmentation provided measurements of central retinal thickness (CRT) and macular volume. CRT was defined as the vertical distance between the vitreoretinal interface and Bruch’s membrane. The total macular volume was calculated using the manufacturer’s software (version 3.1) over a 30° horizontal field of view (approximately 8.8 mm of retinal coverage centered on the fovea). Moreover, the presence of intraretinal fluid (IRF) was evaluated and defined as hyporeflective, well-demarcated cystoid spaces located within the neurosensory retina on SD-OCT B-scans. To provide a comprehensive analysis of structure—function association in RP eyes according to FAF pattern a macular status in SD-OCT was classified based on evaluation made by two independent graders who were masked to clinical data and FAF classification. In cases of disagreement, a consensus was reached by joint review with third external grader. Consequently, macular atrophy was defined as thinning and structural loss of the outer retinal layers on SD-OCT. The epiretinal membrane was defined as a hyperreflective layer on the inner retinal surface, associated with retinal surface wrinkling and/or distortion of the underlying retinal layers. In addition, the cystoid macular edema was defined as the presence of hyporeflective, well-demarcated cystoid spaces within the neurosensory retina. When preserved retinal architecture without evidence of macular atrophy, intraretinal fluid, or epiretinal membrane was detected, RP eye was classified on SD-OCT as macula with no alterations.

In addition, the ellipsoid zone (EZ) was assessed on horizontal foveal OCT scans and classified as intact when a continuous band extending between the nasal and temporal margins and abutting the retinal pigment epithelium (RPE) was clearly identifiable. EZ disruption was defined by an indistinct or discontinuous band. Moreover, in the intact group the EZ length was manually measured using the caliper function. Two independent examiners performed all the measurements. If the difference between the results exceeded 10%, a third examiner repeated the measurement, and the final value was calculated as the mean of all three results to ensure consistency and reproducibility.

### 2.4. Ocular Electrophysiology

To confirm the clinical diagnosis of RP, ERG was performed in all participants using the RetiScan system (Roland Consult, Brandenburg, Germany), in accordance with the standards of the International Society for Clinical Electrophysiology of Vision (ISCEV) [9]. Because the residual visual function in RP is primarily confined to the macular region, a detailed assessment of localized retinal bioelectrical activity was additionally conducted through multifocal electroretinography (mfERG) (RetiScan system; Roland Consult, Brandenburg, Germany). The procedure followed the ISCEV standard for clinical mfERG [10]. Unilateral stimulation was applied using a black-and-white hexagonal array composed of 103 elements. The recording setup included a DTL fibre electrode (Diagnosys LLC, Lowell, MA, USA) as the active electrode, with a gold disc skin electrode (Roland Consult, Brandenburg, Germany) serving as both reference and ground. Each mfERG trace represented the average of six consecutive recordings per eye, with standardized waveform smoothing and manual correction of cursor placement when necessary. The response density (nV/degree^2^) and implicit time (ms) of the P1 wave were analysed within six concentric rings corresponding to increasing eccentricities from the fovea, providing a detailed topographic representation of macular function.

### 2.5. Statistical Analysis

All the statistical analyses were conducted using Statistica software, version 13.3 (TIBCO Software Inc., Palo Alto, CA, USA). The Shapiro–Wilk test was applied to assess the normality of the data distribution. Depending on the data type, comparisons among the three groups defined by the FAF pattern were performed using the Kruskal–Wallis test for nonparametric and the F test (ANOVA) for parametric variables. When significant differences were detected, appropriate post hoc tests were conducted to identify pairwise group differences. For analyses involving two groups with nonparametric distributions, the Mann–Whitney U test was used. Associations between nonparametric variables were evaluated using Spearman’s rank correlation coefficient (Rs), whereas nominal variables were analysed with the maximum likelihood ratio chi-square test. Nominal variables were compared using the chi-square test or Fisher exact test when appropriate. A *p* value less than 0.05 was considered statistically significant. Data are presented as median values with interquartile ranges for variables exhibiting substantial deviation from a normal distribution.

## 3. Results

### 3.1. Subjects

A total of 146 eyes from 73 patients diagnosed with RP were included in the study. The clinical characteristics of the study population are summarized in Table 1. Based on FAF imaging, 76 eyes exhibited an irregular hyperautofluorescent ring, 23 eyes displayed a regular ring, and 47 eyes demonstrated no visible ring. Asymmetrical FAF ring patterns between fellow eyes were observed in 8 individuals. Specifically, 6 patients demonstrated a combination of regular and irregular FAF rings between eyes, while in two patients, discordant patterns were noted, including a combination of regular and absent FAF rings.

**Figure 2:**
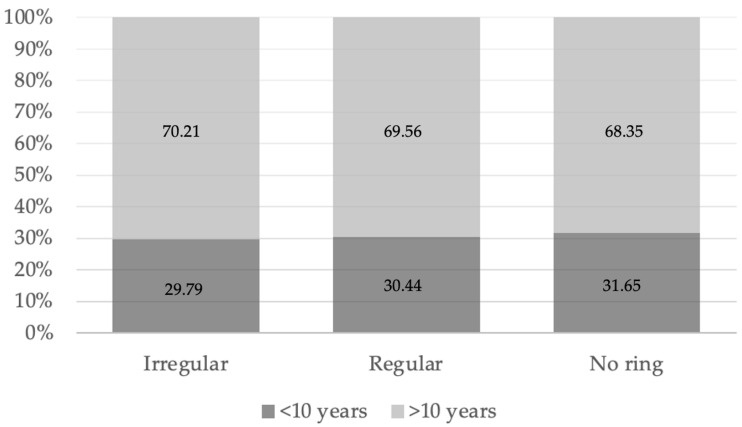
The percentage distribution of symptom duration in retinitis pigmentosa patients according to the fundus autofluorescence pattern.

There were no significant differences among the three FAF groups with respect to age (*p* = 0.068) or sex distribution (*p* = 0.609). Subsequently, we explored the potential associations between the FAF pattern and disease history, symptom duration and genetic background, when available. Because the fellow eyes of the same patient occasionally presented different FAF patterns, each eye was evaluated separately in relation to the clinical history. Accordingly, symptoms persisting for more than 10 years were reported in 33 eyes (70.21%) with an irregular ring, 16 eyes (69.56%) with a regular ring, and 54 eyes (68.35%) without a detectable ring. This distribution did not differ significantly between the groups (*p* = 0.961), suggesting that there was no relationship between disease duration and the FAF pattern (Figure 2). Similarly, no significant differences were observed among the three groups regarding a positive family history of RP (Table 1).

Given the genetic heterogeneity of RP, the inheritance pattern was analysed in available cases. Among eyes with an irregular FAF ring, 2 cases (2.63%) were associated with autosomal recessive inheritance, 6 cases (7.89%) with X-linked inheritance, and 16 cases (21.05%) with Usher syndrome. In the regular ring group, 2 patients (8.69%) had an autosomal recessive mutation, whereas in the group without a ring, 2 patients (4.25%) had an X-linked mutation. Although these distributions did not differ significantly between the FAF groups (*p* = 0.122), the majority of patients had an unknown genetic status.

Additionally, intraocular pressure (IOP) did not differ significantly among the groups (*p* = 0.689).

### 3.2. Functional Analysis

To assess the clinical relevance of FAF patterns in RP, a detailed evaluation of central retinal function was performed across FAF subgroups, as summarized in Table 2.

Eyes with a regular hyperautofluorescent ring had a significantly better BCVA than those with an irregular (*p* < 0.001) or absent ring pattern (*p* = 0.001). Similarly, the contrast sensitivity was markedly higher in the regular ring subgroup than in both the irregular (*p* < 0.001) and absent ring subgroups (*p* < 0.001).

Given the subjective nature and variability of psychophysical measures such as BCVA and contrast sensitivity, these findings were further verified using mfERG to provide an objective assessment of macular bioelectrical activity. The amplitude density in the first mfERG ring, representing the foveal response, was significantly higher in eyes with a regular FAF ring than in those with an irregular ring (*p* = 0.034). A similar trend was observed for the second mfERG ring, where the amplitude density tended to be higher in the regular ring subgroup (*p* = 0.063). As expected in RP, mfERG responses from the outer retinal regions (rings 3–6) were typically undetectable across all subgroups and therefore excluded from further analysis. No significant differences were found among the groups in P1-wave implicit time in either the first or second mfERG ring (Table 2).

Because the most disabling functional impairment in RP is progressive visual field constriction, mean deviation (MD) values from 10-2 and 30-2 static perimeters were also analysed (Table 2). Eyes with an irregular FAF pattern exhibited significantly lower MD values both in the 10-2 and 30-2 groups than those with a regular FAF pattern (*p* = 0.042 and *p* = 0.027, respectively), indicating greater visual field loss.

Collectively, these results demonstrate that a regular FAF pattern in RP is associated with better central vision, higher contrast sensitivity, superior macular bioelectrical activity, and less advanced deterioration of the visual field, reflecting a more preserved retinal structure and function.

### 3.3. Structural Analysis

Next, several morphological parameters were analysed to assess their relationships with FAF patterns. Accordingly, no significant differences in CRT were observed among the three FAF subgroups. Specifically, CRT values did not differ significantly between eyes with irregular and regular rings (250 [99] µm vs. 268 [76] µm; *p* = 0.091), between regular and absent rings (268 [76] µm vs. 276.5 [145] µm; *p* = 0.871), or between irregular and absent rings (250 [99] µm vs. 276.5 [145] µm; *p* = 0.111).

**Figure 3:**
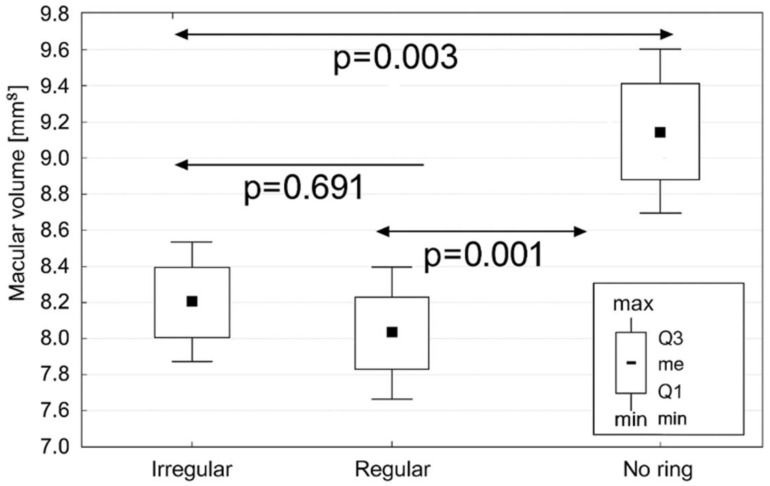
Macular volume in retinitis pigmentosa eyes according to the fundus autofluorescence pattern. Whiskers range from the 10th to 90th percentiles. The square inside the box indicates the median.

However, the analysis of macular volume revealed notable differences. Eyes without a visible FAF ring demonstrated a significantly greater macular volume (8.91 [1.71] mm^3^) than both the regular ring (7.61 [1.28] mm^3^; *p* = 0.001) and irregular ring subgroups (8.06 [1.24] mm^3^; *p* = 0.003) (Figure 3). These findings suggest that area-based volumetric measurements are more sensitive indicators of retinal morphology in RP than single-point thickness assessments. To further explore the observed differences in macular volume between FAF groups, the prevalence of IRF was analyzed. The IRF was detected in 43 eyes (56.58%) with an irregular FAF ring, 5 eyes (21.74%) with a regular FAF ring, and 27 eyes (57.55%) without a detectable ring. Although a tendency toward a higher prevalence of IRF was observed in eyes with irregular or absent FAF patterns, this difference did not reach statistical significance (*p* = 0.082). Moreover, this phenomenon was further explored by offering a detailed analysis in FAF groups according to macular morphology (Table 3). Consequently, the highest proportion of RP eyes without macular alterations was observed in eyes with a regular FAF ring (*p* < 0.001). Contrarily, no significant differences in the prevalence of atrophy (*p* = 0.41) or epiretinal membrane (*p* = 0.23) in RP eyes were observed between FAF groups. Although the prevalence of cystoid macular edema showed a trend toward differences between the FAF group, this did not reach statistical significance (*p* = 0.051).

**Figure 4:**
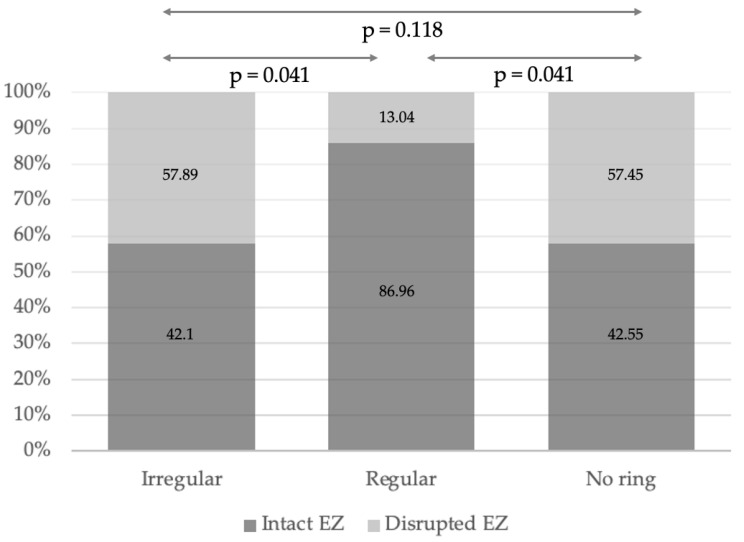
Distribution of the ellipsoid zone width in retinitis pigmentosa eyes according to the fundus autofluorescence pattern.

The EZ was subsequently analysed in relation to FAF patterns. Overall, EZ disruption was identified in 56 RP eyes, including 32 eyes (42.1%) in the irregular ring group, 4 eyes (13.04%) in the regular ring group, and 20 eyes (42.55%) in eyes without a visible FAF ring. A significant difference in EZ morphology distribution was observed among the FAF subgroups. As shown in Figure 4, eyes with a regular FAF ring exhibited an intact EZ significantly more frequently than eyes in the remaining groups (*p* = 0.041). In contrast, disrupted EZ configurations predominated in eyes with irregular or absent FAF rings. In eyes with a regular FAF ring, the intact EZ configuration predominated, whereas disrupted EZ patterns were more frequent in irregular and absent ring groups. Although eyes with a regular FAF ring exhibited a greater EZ width (3103 (1414) µm) compared with eyes with an irregular FAF ring (1934.5 (2581) µm) and those without a visible FAF ring (1962 (5086) µm), this difference did not reach statistical significance (*p* = 0.08). Notably, the irregular and absent FAF ring groups demonstrated substantially wider interquartile ranges, indicating greater heterogeneity of EZ width in these cohorts. In contrast, RP eyes with a regular FAF ring showed a tendency toward a longer and more homogeneous EZ width distribution.

**Figure 5:**
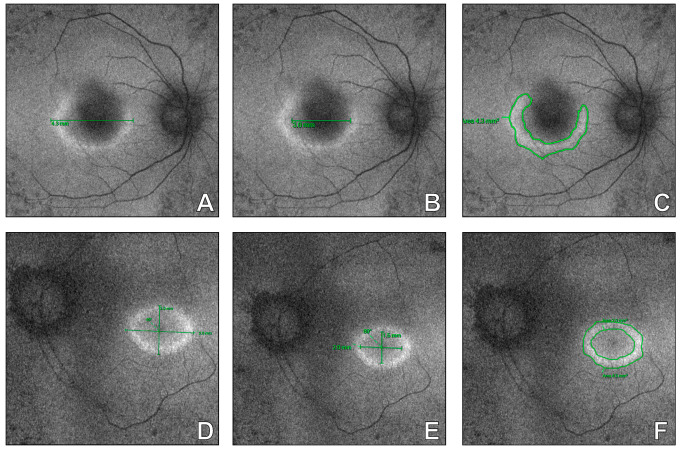
Fundus autofluorescence images of eyes with retinitis pigmentosa showing: irregular pattern of hyperfluorescent ring with external (**A**) and internal (**B**) dimensions, ring area (**C**); regular hyperfluorescent ring with external (**D**) and internal (**E**) dimensions, ring area (**F**).

Next, to enable a comprehensive assessment of FAF ring morphology in RP eyes with regular and irregular patterns, manual measurements of the rings were performed as previously described and illustrated in Figure 5. Notably, in eyes with irregular FAF rings, reliable measurement of both horizontal and vertical dimensions was not feasible in all cases. Since the EZ width is a recognizable structural marker reflecting macular function, a correlation analysis of that parameter with hyperfluorescent ring dimensions was performed (Table 4). Consequently, in RP eyes with a regular FAF ring a significant positive correlation between the EZ length and the hyperfluorescent ring area was detected (Rs = +0.745; *p* = 0.002). Moreover, in this subgroup, a significant positive association was found between the EZ length and macular volume (Rs = +0.587; *p* = 0.005). Importantly, similar correlations in RP eyes with irregular FAF pattern were not observed (Rs = +0.489; *p* = 0.089 and Rs = +0.235; *p* = 0.145, respectively). These findings highlight that FAF morphology reflects macular integrity in RP eyes and might be essential for monitoring of the disease progression.

Together, these results indicate that both a regular FAF pattern and continuous integrity are structural markers of better-preserved central retinal function in RP eyes.

Interestingly, eyes with an irregular FAF pattern demonstrated a significantly larger external ring area as well as a greater total hyperautofluorescent ring area compared with eyes presenting a regular ring (Table 5).

### 3.4. Analysis of Correlations Between Functional and Structural Parameters

Additionally, the relationships between retinal structure and function, as well as correlations between functional parameters and FAF ring dimensions, were analysed. A significant negative correlation was found between the external FAF ring area and contrast sensitivity (Rs = −0.455; *p* = 0.006), indicating that larger hyperautofluorescent rings were associated with poorer contrast sensitivity. These findings support the hypothesis that the spatial extent of hyperautofluorescence corresponds to zones of photoreceptor degeneration.

These observations were further validated by correlating FAF morphometric parameters with objective measures of retinal function. In eyes with a regular FAF pattern, the ring area correlated positively with the mfERG amplitude density in both the first and second rings (Rs = +0.573, *p* = 0.016; and Rs = +0.736, *p* = 0.001, respectively). Moreover, in this subgroup, a significant positive association was found between the total hyperautofluorescent ring area and macular volume (Rs = +0.667; *p* = 0.003).

Collectively, these findings indicate that in RP, a wider hyperautofluorescent ring on FAF imaging is linked to a better-preserved bioelectrical response of the central retina, reflecting residual functional integrity within the photoreceptor layer.

## 4. Discussion

The systematic classification of FAF patterns into three distinct categories, namely regular, irregular, and absent rings, was proposed by Antropoli et al. in 2023 [8]. These authors reported an association between the FAF pattern and structural changes, defined as the EZ width in RP eyes. Based on this classification, we attempt to characterize the central retina according to individual FAF patterns, considering a wide range of morphological and functional measures.

Despite the limited number of studies, there is strong evidence that FAF appearance is closely related to visual acuity in patients with RP [11,12,13,14,15,16]. In the present study, eyes with a regular hyperautofluorescent ring demonstrated significantly better BCVA compared with those showing an irregular or absent ring. Indeed, a regular, symmetrical ring delineates the boundary between the central area of preserved photoreceptors and the surrounding zone of degeneration, justifying higher visual performance in these patients [12,16]. By contrast, an irregular or disrupted ring indicates ongoing degeneration of the outer retinal layers, including the cone photoreceptors, which corresponds to a decline in visual acuity [14,16]. Consequently, the absence of a hyperautofluorescent ring reflects advanced disease stages, where complete loss of photoreceptor integrity in the macula results in profound visual impairment [12,15]. Importantly, the negative correlation between the external FAF ring area and contrast sensitivity observed in our study further confirms that as the hyperautofluorescent ring expands, the functional performance of the central retina declines. These findings indicate a wider zone of photoreceptor stress and lipofuscin accumulation. Notably, contrast sensitivity has never been compared with FAF findings in RP eyes. Consequently, this robust study strengthens the evidence for using FAF imaging as a surrogate biomarker of central retinal function by linking quantitative FAF parameters with functional outcomes.

Owing to the subjective nature of BCVA measurements, the current analysis was supported by an objective investigation of functional parameters of the central retina. Importantly, our results demonstrate a strong association between the FAF pattern and macular bioelectrical activity as assessed by mfERG. Interestingly, RP eyes exhibiting a regular hyperautofluorescent ring showed higher P1-wave amplitude densities in the first mfERG ring, which represented the foveal response. Additionally, a tendency towards higher values in the second ring indicated better preservation of cone function in the central retina. As previously reported, a regular, well-defined FAF ring delineates the transition zone between preserved and degenerated photoreceptors [15,16]. The more compact and symmetrical the ring is, the greater the likelihood of EZ continuity and stronger central P1 responses in mfERG [12,15]. These findings are consistent with previous observations in which irregular or open rings indicated mosaic degeneration of cones and diffuse expansion of lipofuscin-related stress, resulting in attenuated mfERG amplitudes and prolonged implicit times [16,17,18]. Moreover, in our cohort, a positive correlation was observed between the FAF ring area and P1-wave amplitude density in the central mfERG rings, particularly in the subgroup with a regular pattern. Consequently, our findings confirmed a close correspondence between structural and functional markers [12,18]. This structure–function relationship might be particularly valuable for risk stratification, the selection of candidates for experimental therapies, and the definition of sensitive functional endpoints in longitudinal studies of RP [12,15,16,17,18].

The results of the present study revealed that eyes with RP presenting an irregular ring or the absence of a FAF ring exhibited more pronounced visual field defects, defined as less favourable MD values, for both the 10-2 and the 30-2 testing strategies. Indeed, clinically, progressive constriction and relatively preserved central visual islands in eyes with more advanced stages of RP are reflected by more negative MD values [19,20]. According to the literature, quantitative FAF metrics (ring diameters and area) correlate with global perimetric indices, indicating that enlargement or irregularity of the FAF ring is generally associated with decreasing MD values, whereas centripetal constriction of a regular ring may predict imminent deterioration of central threshold sensitivity [12,15,16,18,19,20]. Therefore, the combined assessment using FAF imaging and perimetry provides complementary insight into disease progression.

Further, our study links functional abnormalities corresponding to FAF variants with a comprehensive analysis of the retinal structure. In RP eyes, differences in macular volume reflect several overlapping processes, including macular atrophy, mild retinal or intraretinal oedema, reactive Müller cell gliosis, early tractional changes such as subtle epiretinal membranes, and expansion of extracellular spaces within the outer retinal layers [21,22,23]. A regular FAF ring is usually accompanied by a continuous EZ and is associated with more preserved microstructural integrity. Thus, a smaller macular volume, consistent with better central retinal function, is detected unless it is associated with macular atrophy [20,23,24]. Macular structural abnormalities are common in retinitis pigmentosa and substantially contribute to heterogeneity of central retinal morphology. As demonstrated by Nebbioso et al. [25], cystoid macular edema and epiretinal membrane are frequent OCT findings in RP and may lead to increased retinal thickness despite ongoing photoreceptor degeneration. This observation is consistent with our results, in which eyes without a detectable FAF ring showed the highest macular volume, likely reflecting a higher prevalence of such secondary structural changes. To advocate his hypothesis, in this study a trend towards higher prevalence of IRF and cystoid macular edema in RP eyes with irregular and no FAF ring, as well as significantly more cases of lack of macular alterations in eyes presenting regular hyperfluorescent ring, were noted. That might suggest that FAF ring configuration mirrors the underlying photoreceptor status and disease stage. These findings highlight the importance of integrating FAF imaging with detailed OCT-based macular assessment when interpreting structure–function relationships in RP. Therefore, from a clinical perspective, combined FAF and OCT assessment may provide a practical tool for monitoring disease progression and stratifying patients with RP according to the risk of central retinal involvement [25]. Importantly, this is the first study revealing that macular volume differentiated FAF-based subgroups more distinctly than CRT. These findings suggest that area- or volume-based parameters are more sensitive indicators of retinal architecture alterations in RP than single-point measurements taken at the fovea. This combined approach may enhance the early detection of disease progression, help distinguish stable degenerative changes from potentially reversible (e.g., oedematous) components, and support individualized decisions regarding treatment and follow-up intervals. Notably, the absence of a FAF ring should prompt careful assessment of macular volume even when it appears normal, as volumetric metrics may represent an earlier and more sensitive indicator of structural deterioration in RP.

Interestingly, the findings of the present study confirm a strong association between the FAF pattern and the continuity of the EZ in eyes affected by RP. The EZ band represents the ordered arrangement of mitochondria within the photoreceptor inner segments and serves as a sensitive indicator of photoreceptor viability [14,26]. In eyes with a regular hyperautofluorescent ring, the EZ band appeared continuous and well preserved, indicating that the integrity of the cone photoreceptor outer segments was maintained. By contrast, eyes with an irregular ring or the absence of a ring frequently exhibited fragmentation or complete loss of the EZ, corresponding to advanced photoreceptor degeneration and reduced central retinal function. This relationship aligns with the findings of previous studies reporting a positive correlation between FAF ring diameter and residual EZ width, both in cross-sectional and longitudinal analyses [14,15,23]. From a clinical standpoint, combined evaluation of FAF and OCT-derived EZ integrity enables precise mapping of the transition zone, providing a robust tool for monitoring disease dynamics, identifying candidates for emerging therapies, and evaluating treatment efficacy in clinical trials [14,15,23,25,26].

Nevertheless, several limitations should be acknowledged. First, a potential selection bias related to a floor effect cannot be excluded. Due to the prospective enrollment strategy and the reliance on several subjective measures of visual function, individuals with advanced RP who were unable to reliably perform BCVA, contrast sensitivity, or perimetry testing were not included. Although this approach was necessary to meet the comparative aims of the study, it may limit the generalizability of the findings to the most severely affected patients. Second, incomplete characterization of the genetic background in a substantial proportion of participants precluded meaningful genotype–phenotype correlations, which may contribute to variability in FAF patterns. Importantly, all included individuals exhibited typical fundus features of RP, and the diagnosis was confirmed by ERG. Third, although retinitis pigmentosa is a genetic disorder with bilateral involvement, asymmetrical retinal manifestations between fellow eyes may occur. Consequently, eye-level rather than individual-level grouping was required in this study due to FAF ring asymmetry. This approach limits direct interpretation of FAF pattern prevalence in relation to person-based variables such as sex, age, or family history. However, given the structure–function focus of the present analysis, eye-level assessment is considered appropriate. Finally, the absence of longitudinal follow-up precluded assessment of temporal changes in FAF morphology and their relationship with progressive functional decline. Longitudinal studies addressing these dynamics may represent an important direction for future research.

## 5. Conclusions

To the best of the authors’ knowledge, this is the largest study to date to comprehensively evaluate functional and structural changes in the central retina in RP eyes using a modern FAF-based classification. Consequently, the present study identifies FAF-derived biomarkers that reflect disease severity and retinal integrity. Accordingly, the spatial extent and regularity of the hyperautofluorescent ring correspond directly to preserved visual function and macular bioelectrical activity. In particular, larger and more regular FAF rings were associated with higher BCVA, better contrast sensitivity, and more mfERG responses. Thus, FAF imaging and its morphometric analysis may serve as a valuable substitute for long-lasting and wearable mfERG and perimetry testing or as a useful tool for precise monitoring of disease progression when combined with multimodal assessments.

## Figures and Tables

**Table 1:** Characteristics of patients with retinitis pigmentosa according to fundus autofluorescence pattern.

Parameter	Type of a Hyperfluorescent Ring			* *p* Value
	Irregular	Regular	No Ring	
Total number	76	23	47	
Sex: Female	34	13	24	0.609
Male	42	10	23	
Age [years]	40 (25)	37 (17)	42 (23)	0.068
Median (IQR)				
RP positive family history [%]	13.16	0	6.38	0.072
IOP [mmHg]	16 (4)	16 (4)	15.5 (4.4)	0.689
Median (IQR)				

Abbreviations: RP, Retinitis pigmentosa; IQR, interquartile range; IOP, Intraocular pressure. * Chi-square test or Fisher exact test for nominal variables. Kruskal–Wallis test for continuous and nonparametric variables.

**Table 2:** Comparison of functional eye parameters in retinitis pigmentosa patients according to fundus autofluorescence pattern.

Parameter	Type of a Hyperfluorescent Ring			* *p* Value		
	Irregular (1)	Regular (2)	No Ring (3)	1 vs. 2	2 vs. 3	1 vs. 3
BCVA [ETDRS, No. of letters]	17 (23)	33 (18)	18 (26)	**<0.001**	**0.003**	0.986
Contrast sensitivity [No. in Pelli-Robson]	18 (11)	24 (6)	17 (12)	**<0.001**	**<0.001**	0.757
Amplitude density in first ring in mfERG [nV/degree^2^]	33 (31)	50 (45)	41 (34)	**0.034**	0.669	0.486
Culmination time in first ring in mfERG [ms]	44.10 (8.80)	44.10 (7.90)	44.10 (13.7)	0.964	0.936	0.744
Amplitude density in second ring in mfERG [nV/degree^2^]	11.92 (14.05)	17.15 (14.64)	14.21 (12.25)	0.063	0.309	0.701
Culmination time in second ring in mfERG [ms]	41.20 (7.80)	43.10 (8.80)	44.10 (13.7)	0.136	0.842	0.297
10-2 MD	−19.96 (11.72)	−14.23 (16.52)	−24.37 (17.69)	**0.042**	0.281	0.361
30-2 MD	−29.88 (5.45)	−26.66 (9.18)	−28.05 (13.31)	**0.027**	0.403	0.311

Abbreviations: BCVA, Best corrected visual acuity; ETDRS, Early Treatment Diabetic Retinopathy Study; mfERG, multifocal electroretinography; MD, Mean Deviation. * Kruskal–Wallis test with post hoc analysis. Significant values are shown in bold. Data are presented as median values (interquartile ranges).

**Table 3:** Characteristics of macular morphology in optical coherence tomography of eyes with retinitis pigmentosa according to fundus autofluorescence pattern.

Macular Status	Type of a Hyperfluorescent Ring			* *p* Value
	Irregular	Regular	No Ring	
No alterations	18 (23.68%)	17 (73.91%)	10 (21.28%)	**<0.001**
Atrophy	15 (19.74%)	2 (8.69%)	10 (21.28%)	0.41
Epiretinal membrane	20 (26.32%)	4 (17.39%)	17 (36.17%)	0.23
Cystoid macular edema	10 (21.28%)	0 (0%)	10 (21.28%)	0.051

* Chi-square test or Fisher exact test for nominal variables. Significant values are shown in bold.

**Table 4:** Correlation of the ellipsoid zone length and dimensions of autofluorescence pattern in retinitis pigmentosa eyes according to type of hyperfluorescent pattern.

Correlation of the Ellipsoid Zone Length and:	Type of a Hyperfluorescent Ring	
	Irregular	Regular
Diameter [mm]		
Internal		
Horizontal	**+0.873**	**+0.669**
Vertical	**+0.762**	**+0.588**
External		
Horizontal	**+0.679**	**+0.561**
Vertical	**+0.682**	**+0.763**
Area [mm^3^]		
Internal	**+0.829**	**+0.704**
External	**+0.696**	**+0.763**
Hyperfluorescent ring	+0.489	**+0.745**

Spearman’s rank correlation coefficient (Rs). Significant values are shown in bold.

**Table 5:** Comparison of dimensions of autofluorescence pattern in retinitis pigmentosa eyes.

Parameter	Type of a Hyperfluorescent Ring		* *p* Value
	Irregular	Regular	
Diameter [mm]			
Internal			
Horizontal	2.5 (7.4)	2.35 (1.15)	0.991
Vertical	2.3 (2.7)	1.75 (0.8)	0.623
External			
Horizontal	5.85 (7.05)	3.9 (1.15)	0.098
Vertical	4.1 (4.15)	2.75 (0.85)	0.101
Area [mm^3^]			
Internal	3.2 (13.3)	2.65 (3.7)	0.682
External	22.3 (35.8)	7.9 (6.3)	**0.014**
Hyperfluorescent ring	13.75 (29.85)	2.35 (1.15)	**0.016**

* Mann–Whitney U test. Significant values are shown in bold. Data are presented as median values (interquartile range).

## Data Availability

The data used to support the findings of this study are available from the corresponding author upon request.

## Abbreviations

The following abbreviations are used in this manuscript:

RP	Retinitis pigmentosa
FAF	Fundus autofluorescence
RPE	Retinal pigment epithelium
ERG	Full-field electroretinography
BCVA	Best-corrected visual acuity
SD-OCT	Spectral-domain optical coherence tomography
CRT	Central retinal thickness
EZ	Ellipsoid zone
ISCEV	International Society for Clinical Electrophysiology of Vision
mfERG	Multifocal electroretinography
MD	Mean deviation
IRF	Intraretinal fluid
